# Septic acute kidney injury: a review of basic research

**DOI:** 10.1007/s10157-020-01951-3

**Published:** 2020-08-11

**Authors:** Daisuke Nakano

**Affiliations:** Department of Pharmacology, Faculty of Medicine, Kagawa University, 1750-1 Ikenobe, Miki, Kita, Kagawa 761-0793 Japan

**Keywords:** Acute kidney injury, Sepsis

## Abstract

Sepsis is a major cause of acute kidney injury (AKI) among patients in the intensive care unit. However, the numbers of basic science papers for septic AKI account for only 1% of all publications on AKI. This may be partially attributable to the specific pathophysiology of septic AKI as compared to that of the other types of AKI because it shows only modest histological changes despite functional decline and often requires real-time functional analysis. To increase the scope of research in this field, this article reviews the basic research information that has been reported thus far on the subject of septic AKI, mainly from the viewpoint of functional dysregulation, including some knowledge acquired with multiphoton intravital imaging. Moreover, the efficacy and limitation of the potential novel therapies are discussed. Finally, the author proposes several points that should be considered when designing the study, such as monitoring the long-term effects of the intervention and reflecting the clinical settings for identifying the molecular mechanisms and for challenging the intervention effects.

## Introduction

**Fig. 1:**
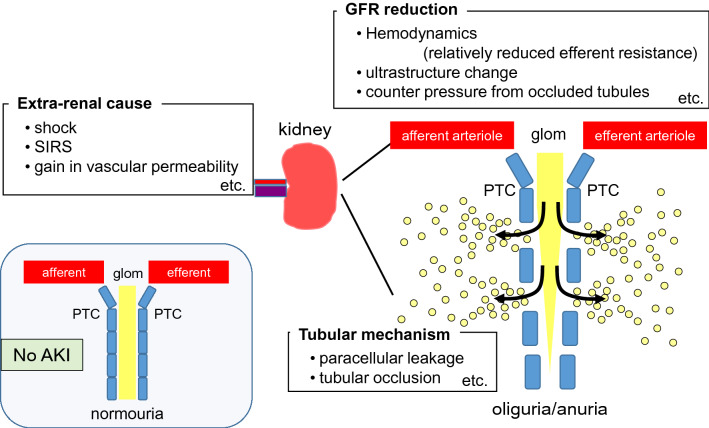
Causes of septic oliguria/anuria. Fluid resuscitation could resolve dehydration and glomerular filtration rate (GFR) reduction, but not tubular leakage (depicted by small yellow circles). Rather, continuous fluids under tubular leakage accelerate a positive balance. GFR reduction and tubular occlusion could deteriorate each other

Acute kidney injury (AKI) is an important challenge that negatively impacts patient survival in an intensive care unit (ICU). Although there was a 9% decline in the in-hospital mortality rate among patients treated with renal replacement therapy in the ICU from 2007 (44.9%) to 2016 (36.1%) in Japan, the mortality rate of sepsis patients remains high at > 50% [1]. Moreover, translational research for AKI has not yet been successful, and no specific treatment has been established in the clinical setting. The search result for the keywords “acute, kidney, injury, and rats” in PubMed showed over 6000 scientific articles, and “mice” instead of “rats” provides an additional above 3800 papers (June 2020). Sepsis is a major cause of AKI; however, when the term “sepsis” was added to either search, it decreased the results to < 270 (< 5%) and < 400 (< 11%), respectively. These numbers (rats + mice) account for about only 1% of all AKI publications. Septic AKI can lead to mortality in patients in the intensive care; therefore, this topic is relevant and should focus on more basic research. Septic AKI involves an infection that critically influences the development of AKI as compared to the other types of AKI. Thus, this review aimed to distinguish septic AKI from other types of AKI, discuss the basic scientific outcomes based on functional changes (Fig. 1), and introduce potential novel treatments.

## Reduction in the glomerular filtration rate (GFR)

### Glomerular hemodynamics

Changes in the GFR, from the viewpoint of hemodynamic change, are currently investigated on relatively big animals, such as dogs and sheep, because it is easier to create ICU-like experimental settings for these animals as compared to that for smaller animals, such as rodents, and to perform continuous inulin/inulin-like compound-used clearance analyses. The former may be essential for the replication of a hyperdynamic state-like human septic AKI [2]. Changes in the cardiac output were accompanied by changes in the renal blood flow in septic animals [3]. Thus, animals in the hyperdynamic states typically show increased renal blood flow despite reduced renal function in septic AKI. One hypothesis for decline in GFR under conditions of increased renal blood flow is the gain in the ratio of the efferent-to-afferent arteriole diameter under efferent vasodilation that increases plasma flow to the peritubular capillaries. The vasoconstrictors that showed higher affinity for the efferent arterioles, such as angiotensin II, might prevent AKI in the animals; thus, this is an area of focus in human AKI research [4, 5]. It is noteworthy that angiotensin II infusions initiated at 2 h after *Escherichia coli* infusions ameliorated both oliguria and serum creatinine levels, despite reduced renal blood flow in sheep [6]. Studies on sheep or pigs also reported that angiotensin II did not worsen the ATP level [7], medullary hypoxia [8], and mitochondrial respiration [9] during sepsis, suggesting that the vasoconstrictive effects of angiotensin II on the efferent arteriole might not induce reductions in blood supply to the peritubular capillaries. In a rodent sepsis model, lipopolysaccharides (LPS), an endotoxin, introduced at a dosage designed to induce AKI, constricted the afferent arteriole and decreased the ratio of the efferent-to-afferent arteriole diameter [10], thereby reducing the GFR.

### Changes in glomerular cells

In addition to the afferent/efferent arteriole-dependent intraglomerular pressure changes, Xu et al. [11] reported that endotoxemia caused ultrastructural alterations in the glomerular endothelium. The endothelial surface layer (ESL), also called the glycocalyx, is a barrier composed of negatively charged proteoglycans and glycoproteins. The ESL covers the endothelial fenestrae and limits the proteins from permeating the fenestrae; however, breakdown of this layer promotes permeability and allows access to even relatively large proteins, such as albumin [12]. Xu et al. [11] reported that endotoxemia produced TNF-α that degraded the ESL in the glomerular endothelium that was consistent with the observations reported in the vasculature of other organs [13]. Furthermore, LPS/TNF-α induced glomerular endothelial swelling and fenestrae density reduction at 24 h. Although an increase in albumin filtration increases colloidal osmotic pressure elevation in the Bowman’s capsule, the “benefit” of the GFR might be imperceptible owing to the fenestrae alteration that occurs during endotoxemia. The physiological turnover rate of the ESL components was approximately 5 days [14]; thus, the alterations in the glomerular endothelium were expected to be reversible after the end of the cytokine storm.

### Prerenal cause

The prerenal causes of GFR reduction were often resolved by fluid and vasopressor treatment although this might not be the case if sepsis increased the systemic vascular permeability. The optimization of fluid resuscitation for sepsis or septic AKI in clinical studies has been debated in multiple review articles [15–19]. However, few studies have compared the efficacy of fluid use in septic AKI in basic studies [20–24]. Most interventional studies against septic AKI, especially those performed on rodents did not reflect a clinical setting because a small animal ICU setting is difficult to create/maintain.

## Reduction of tubular flow rate

**Fig. 2:**
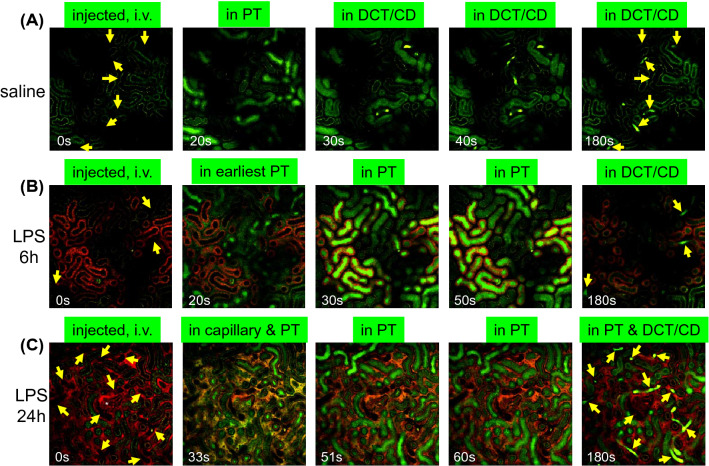
Time-lapse imaging after an intravenous bolus injection of Lucifer yellow, a fluorophore that is freely filtered from the glomeruli. Lucifer yellow was injected into a mouse that had received saline or LPS at either 6 or 24 h earlier. Green fluorescence at 0 s in the image is autofluorescence derived from proximal tubules. Distal nephron (indicated by yellow arrows) does not produce detectable green autofluorescence. **a** Several proximal tubules showed Lucifer yellow in the lumen within 20 s after injection. Lucifer yellow flowed into some distal nephrons at 30 s. At 180 s, Lucifer yellow flowed out from some distal nephrons. **b** Atto 565-conjugated LPS (red) was injected in the mouse used in these images. Atto565-LPS accumulated in a part of the proximal tubules, presumably at the S2 segment. Several proximal tubules showed Lucifer yellow in the lumen within 20 s after injection in the mice 6 h after LPS injection. The inflow rates of Lucifer yellow into these proximal tubules were similar to that in healthy control mice, indicating preserved GFR in these nephrons. At 50 s after injection, Lucifer yellow-derived fluorescence was detected in the proximal tubules that took up LPS (red), but not in distal nephron in the imaging window. Lucifer yellow was detected in the distal tubular lumen at 180 s after the injection. **c** Atto 565-conjugated albumin (red) was injected in the mouse used in these images. The Atto 565-albumin was detected in the peritubular capillaries and glomerulus (white asterisk). At 33 s after following the Lucifer yellow injection 24 h after LPS injection, Lucifer yellow was detected in the Bowman’s space of glomerulus, some proximal tubular lumens, and peritubular capillaries. The slower appearance of Lucifer yellow and more flow into the peritubular capillary, compared to higher images, suggest poor hemodynamics, including GFR reduction. Male C57/BL6J mice were purchased from CLEA (Tokyo, Japan) and housed in our colony until they were of proper age (> 55 week-old) for the induction of AKI consistently. The detailed intravital imaging setting, including microscopy setting, has been described previously [29, 31, 107]. *PT* proximal tubules, *DCT/CD* distal convoluted tubules, and cortical collecting duct

**Fig. 3:**
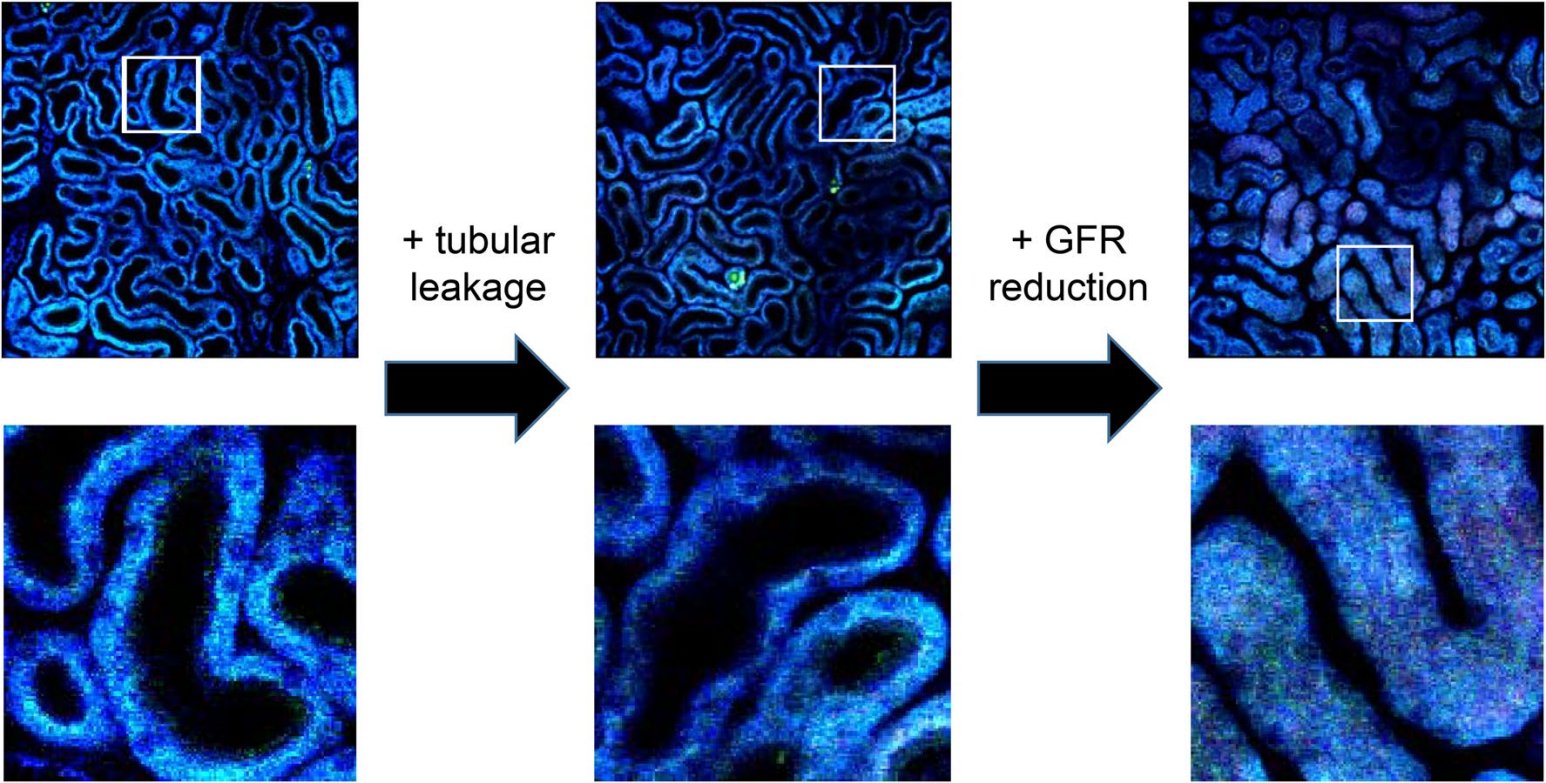
Representative multiphoton unlabeled images of the murine kidney (720-nm excitation laser wavelength) in each stage of septic AKI in the mice aged > 55 week. The left image is from a healthy control mouse. The lower images were enlarged images of the ensquared part in each upper image. The tubular lumen is visible as a dark lumen in each “blue” tubule. The blue color was derived from the autofluorescence of the tubules. There are some bright spots containing strong green fluorescence in the image in the middle; these could be derived from the non-degradable metabolites in the aged tubules (the mouse was 57-week-old). The middle is from a mouse that received LPS (5 mg/kg) 6 h before image acquisition. The tubular lumen is visible, while the tubular flow rate is slowed (see images in Fig. 2b). The single nephron GFR in the mouse was at similar levels as those in LPS-untreated normal mice that were evaluated based on how quickly the Lucifer yellow dye flowed into the first segments of the proximal tubules. The right image is from a mouse that received LPS (5 mg/kg) 24 h before image acquisition. The mouse showed reduced GFR, and the tubular lumen was almost occluded in the image. Male C57/BL6J mice were purchased from CLEA (Tokyo, Japan) and housed in our colony until they were of proper age (> 55 week-old). The detailed intravital imaging setting is described in our previous publication [29, 31, 107]

AKI could occur with oliguria and without a rise in the serum creatinine level. The unresponsiveness of serum creatinine could result from the dilution of creatinine by fluids [25, 26] or via reduced production of creatinine during sepsis [27]. However, the underlying mechanism of development of oliguria in spite of the maintenance of renal circulation with fluid resuscitation and additional vasoactive agents was unknown until recently. We addressed this question using intravital imaging combined with multiphoton microscopy to find that that the filtrate flow rate had reduced in the proximal tubules, while the GFR was maintained at a normal level (Figs. 2 and 3) because of the paracellular leakage of the tubular fluid into the interstitium in rats and mice in the LPS model. The LPS bound to TLR4 in the proximal tubules and subsequent signaling disrupted the tight junctions among the proximal tubular cells, resulting in paracellular leakage [28, 29]. The following experiments revealed that leakage at the proximal tubules affected recovery from oliguria when renal circulation was maintained with fluid resuscitation [29]. The GFR was restored through fluid resuscitation; however, tubular flow did not reach the bladder because of leakage at the proximal tubules (Fig. 1). Mechanistically, LPS/TLR4 stimulated p38 MAP kinase (transiently; < 6 h) and NF-κB (relatively continuously; until 24 h), disrupting the localization of ZO-1 and claudin 2 and reducing occludin expression [29]. Intravenously injected LPS conjugated with fluorophore first (− 20 min) was bound to S1 segments of the proximal tubules [30, 31]; thereafter, it was distributed to both the S1 and S2 segments overtime (1 h) [31]. In a later phase (6, 24, or 48 h), the distribution of the LPS showed heterogeneity; some proximal tubules accumulated LPS, and the others exhibited mild accumulation. However, the cause of heterogeneity remains unclear. These tubules with considerable accumulation of LPS might be downstream S2 segment tubules; however, until now, there was no definitive examination for this speculation. Some tubules highly accumulated LPS, while others showed a reduction in the tubular flow rate or cell swelling [31], resulting in tubular occlusion and halted tubular flow. Continuous intravenous fluid infusion occasionally reopened the occluded tubular lumen that was accompanied by the delivery of tubular fluid to the lumen of the distal nephron, indicating recovery of the tubular flow [31]. Hato et al. [32] reported with elegant fluorescence lifetime microscopy that LPS changes the nucleotide metabolism in the S2 segments of the proximal tubules with increased reactive oxygen species production that was possibly induced by LPS binding to the S1 segment. It is noteworthy that fluorescence lifetime image analysis showed that LPS decreased the metabolic heterogeneity between S1 and S2, albeit with heterogeneity in the tubular flow rate.

## Microcirculation failure

**Fig. 4:**
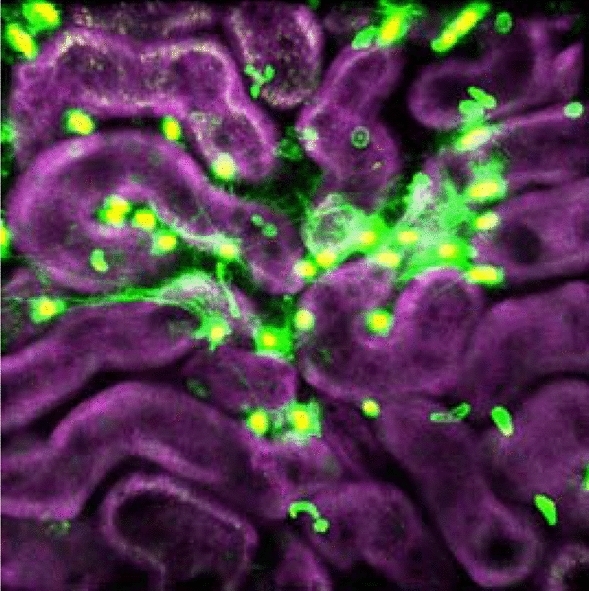
A 3D image of the neutrophil extracellular entrapped in the live kidney of a mouse that received LPS 10 min before capturing. Green represents released/dead cell DNA (Sytox green), and magenta represents the autofluorescence of the tubules. Saturated Sytox green fluorescence changes to yellow in the nuclei. Male C57/BL6J mice were purchased from CLEA (Tokyo, Japan) and housed in our colony until they were of proper age (12 week of age). The image was acquired as previously described [114, 115]

The renal capillary network was often destroyed during non-septic AKI [33], and angiogenesis was indispensable for efficient tubular recovery [34, 35]. In septic AKI, microcirculation failure, rather than histological capillary destruction, caused changes in the oxygen supply to the tubules. Rodent septic AKI models elicited heterogeneity of the red blood cells (RBCs) in each capillary, irrespective of the total renal perfusion or GFR [22, 31, 36]. The microcirculation failure in each capillary could be intermittent or could last for > 10 s [36]. There appeared to be several causes for RBC flow cessation, such as clotting, leukocyte attachment [31], and neutrophil extracellular entrapment (Fig. 4). The capillary occlusion or cessation of the RBC flow-induced reactive nitrogen species production and mitochondrial membrane potential reduction in the adjacent tubules of the endotoxemic rodents [31, 36]. Intravital imaging of the multiphoton laser microscopy carefully analyzed the co-localization between the microcirculation failure of the peritubular capillaries and LPS-accumulated “leaking” in the proximal tubules; however, the occurrence in the capillary network was random [31].

## Histopathological changes

As noted above, AKI is currently diagnosed via the GFR or urine output, and it is important to ensure patient survival during the acute phase. The pathological features of AKI differed, depending on the AKI cause. Renal ischemia/reperfusion injury that is widely employed as an AKI model induces dramatic changes in the renal histology. Conditions related to renal ischemia/reperfusion injury include acute tubular necrosis, cell sloughing, cast formation, perivascular inflammation [37], capillary rarefaction [35], tertiary lymphoid tissue formation [38], tubular ferroptosis [39], and intrarenal denervation [40]. However, these pathological features were not always observed in septic AKI, wherein only modest histological changes in the kidneys were observed in human sepsis and animal models of sepsis [41, 42].

## Therapeutic candidates against septic AKI

Standard supportive treatments, such as fluids and vasoconstrictors, are used to restore the GFR; however, they do not address the changes in the tubules in the septic AKI cases. In addition, there are concerns surrounding fluid resuscitation [43–47]. Therapies that specifically target the reduction in the GFR/tubular flow rate and tubular injury in AKI do not currently exist in clinical settings, although several basic research papers have reported on their efficacy. The problems regarding this failed translation include physical barriers in the laboratory that are not discussed in this review [48]. Instead, this review evaluates several candidate treatments that target relatively multiple factors.

### Ischemic preconditioning

Ischemic preconditioning is a possible strategy against perioperative end-organ damage, including AKI. Repeated short-time hypoxic/ischemic stress to the kidney has caused a tolerance to AKI in multiple experimental models, including sepsis [49–51], ischemia/reperfusion [52–54], and cisplatin models [55] in rodents. This method did not always work for large animals, such as dogs [56] and pigs [57–59]. Remote ischemic preconditioning involves repeated occlusion of the limbs and might be applicable to both, basic and clinical studies. However, the effect was inconsistent; for example, it showed beneficial effects on sepsis in mice [60] and sheep [61], but not on ischemia/reperfusion in mice [62] and pigs [63]. Moreover, the causes of AKI did not appear to affect the results. Remote ischemic preconditioning before LPS injection did not ameliorate decreases in blood pressure, systemic cytokine storms, or the urinary concentration of the tissue inhibitor of metalloproteinases-2*insulin-like growth factor-binding protein-7 in a single-center RCT [64]. Conversely, remote ischemic preconditioning before the induction of anesthesia safely improved the long-term kidney function after living-donor renal transplantation in a double-blind RCT [65].

### Endotoxin preconditioning

Endotoxin preconditioning involves the injection of a single low-dose endotoxin, including LPS, to protect the organs from subsequent events. Experiments on pigs have reported that endotoxin preconditioning suppressed the systemic oxygen demand despite unchanged global hemodynamics in response to the *Salmonella abortus equi* endotoxin [66]. Although the precise mechanism is yet to be clarified, studies on mice have demonstrated that the macrophages were the key cell type required to induce the anti-inflammatory phenotype [67]. Moreover, the protection of the kidneys involved inducing macrophage clustering around the Bowman’s capsule and the S1 segment of the proximal tubules following LPS administration in mice [62]. The beneficial effects of the endotoxin preconditioning largely relied on changes in the macrophage primarily focused on the anti-inflammatory phenotype; therefore, it has been reported that endotoxin preconditioning prevents organ damage associated with inflammation in several organs, such as ischemia-induced neuronal damage [68, 69] and pancreatic damage [70]. However, owing to the modification effects of immune cell function, there are reports of deteriorating disease conditions during sepsis [71] and lupus nephritis [72]. Chen et al. [71] demonstrated that super-low-dose LPS (ng order per kg body weight) exacerbated bacteremia and mortality and reduced neutrophil extracellular trap formation after cecum ligation and puncture in mice. Moreover, low-dose LPS (μg order per kg body weight) improved bacteremia and mortality rate and induced the neutrophil extracellular trap. Currently, no human studies have examined the effects of endotoxin preconditioning on sepsis/AKI because there are multiple concerns and steps that need to be overcome in basic research before clinical application. For example, the LPS dosage used for the human studies was 1–2 ng/kg that was considered a “super-low” priming dosage. The fact that a rodents’ response to LPS was much weaker than that of a human further complicates this issue. Another problem was the influence of risk factors on the protective effects, given that most studies have used young animals, and an older immune response could be different [73]. For instance, a comparison between a 2-month-old and a 12-month-old ICR mice showed that the beneficial effects of endotoxin preconditioning were weaker in the older mice [74].

### Cholinergic anti-inflammatory pathway stimulation

Metz et al. developed a concept that has been termed the cholinergic anti-inflammatory pathway [75], mediated in a β2 adrenergic/α7 nicotinic acetylcholine receptor-dependent manner in the spleen [76, 77]. Several strategies have been used to stimulate the cholinergic anti-inflammatory pathway [78–83]. Although the precise neuronal network responsible for each stimulation remains debated, there are multiple neuronal networks capable of inducing the cholinergic anti-inflammatory pathway, such as the central C1 neurons [82], the peripheral vagus [81, 83, 84], the sympathetic [85], and the sciatic nerves [86]. Nicotine was administered for reno-protection; it stimulated this pathway and protected the kidneys from ischemia/reperfusion injury in mice [87]; however, this treatment could worsen the survival rate during sepsis owing to the suppression of the host defenses [84, 88–90]. Nicotine administration was considered too risky for humans. Several methods were alternatively proposed to stimulate the cholinergic anti-inflammatory pathway. First, electrical vagus nerve stimulation could be performed via a portable device that has already been developed and is awaiting clinical application. The efficacy of vagus nerve stimulation on experimental AKI has been confirmed using the ischemia/reperfusion [81] and cisplatin [91] models. Either afferent or efferent nervous stimulation attenuated AKI via which both nerves were involved in the splenic α7 nicotinic acetylcholine receptor-dependent pathway (although afferent seemed to induce protection partially through an unknown pathway). Second, noninvasive ultrasound exposure reportedly stimulates the peripheral neurons and induces the cholinergic anti-inflammatory pathway. The features of the ultrasonography examination could be used for application to the targeted organ at a variable strength [92]. Splenic ultrasonography modulation suppressed LPS-induced reduction of noradrenaline and acetylcholine and cytokine production expression in the spleen [92] and attenuated AKI in the cecum ligation and puncture [79] and ischemia/reperfusion [83] models. Third, electroacupuncture could be another tool to stimulate this pathway. Electroacupuncture at ST36 acupoint, known as Zusanli, induced anti-inflammatory responses [86] and protected the kidneys in septic models [93, 94]. This protection was attenuated by treatment with reserpine [86, 93], D1 receptor antagonist [86], α7nAChR antagonists [95–98], vagotomy [95, 99], and adrenalectomy [93], but not splenectomy [86]. Therefore, electroacupuncture appeared to induce this protection via the cholinergic and adrenergic pathways.

### Atrial natriuretic peptide

Atrial natriuretic peptide (ANP) is secreted from the atrium following reduced cardiac function. Endogenous ANP secretion is increased during human septic shock [100] and in the ovine hyperdynamic endotoxin model [101]. Its recombinant peptide at a low dosage was used against AKI, based on empirical observations. However, this effect has not been confirmed with strong evidence from multicenter, high-quality, large-sample RCTs [102]. In rodents, ANP suppressed renal ischemia-/reperfusion-induced [103–106] and LPS model injuries [107]. We employed a suppressor dosage of ANP combined with fluid resuscitation in the LPS model of rats and demonstrated that a 2-h treatment with ANP during the early phase (2–4 h after LPS) improved urine flow, GFR, tubular flow leakage, and survival rate [107]; however, the efficacy considerably lowered with treatment in a relatively later phase (18–20 h after LPS). This study also revealed that, in fluid-administered mice, endogenously secreted ANP stimulated its receptor GCA in the endothelial cells and prevented vascular permeability gain and GFR reduction after LPS; further, exogenously administered ANP stimulated GCA in the proximal tubular cells and prevented LPS-induced tubular leakage. The limitations in basic research involved the use of young rodents and the sole purpose of the LPS model. ANP (the human recombinant type used in LPS study) is already approved and has been used for acute heart failure treatment in Japan for the previous 25 years.

### Meditation, exposure to cold, and breathing techniques

Pickkers et al. performed several studies using the endotoxin challenge in humans [108–111] and demonstrated that a training program, composed of meditation, cold exposure, and special breathing, increased adrenaline/noradrenaline in the plasma and created a tolerance against experimental endotoxemia [110]. These data reported the sympathetic nervous system (plasma adrenaline and noradrenaline) stimulation, increased leukocytes in the plasma, acute respiratory alkalosis (normalized immediately after cessation of the special breathing), relatively high lactate, and decreases in oxygen saturation in the trained individuals following LPS administration. The limitation of this method is that the training protocol is too difficult for subjects with AKI risk factors. From the article [110], the following are the examples of this difficulty: “standing in the snow barefoot for up to 30 min and lying bare-chested in the snow for 20 min; daily dipping/swimming in ice-cold water (0–1 °C) for several minutes (including complete submersions); and hiking up a snowy mountain (elevation: 1590 m) bare-chested, wearing nothing but shorts and shoes at temperatures ranging from − 5 to − 12 °C (wind chill: − 12 to − 27 °C)” [110].

The current limitations of these therapeutic candidates are summarized in Table 1.

**Table 1:** Limitations of each therapeutic candidate for human application

	Limitations
Ischemic preconditioning	Efficacy differs by cause of AKI or among species
Endotoxin preconditioning	The optimum dosage window for the endotoxin must be used; if the dosage is too low, it induces priming and may further deteriorate sepsis Aging may weaken the efficacy
Cholinergic anti-inflammatory pathway stimulation	Efficacy of the post-AKI treatment has not yet been examined Aging may weaken the efficacy
ANP	Must be subdepressor dosage Lack of evidence from sepsis model
Cold exposure	The training program is too rigorous Lacks efficacy with sepsis and not an LPS model

## Conclusion and message

Septic AKI is associated with considerable hospital-based mortality; once a novel treatment method has been developed, it could save thousands of lives. Recent developments using artificial intelligence have enabled the prediction of “new” antibiotics [112]. However, this might not be the case for AKI because there is lack of knowledge for the formation of a specific drug/chemical structure to train artificial intelligence, necessitating further basic research. For future basic studies to fight against septic AKI, the following three points should be considered in the study design.

- As much as possible, studies to find the pathogenesis/molecular mechanism should additionally examine the effect of factors existing in the clinical setting. Young rodent tests might show different results than older mice. Use of rodents might benefit the modification of genes (and are less expensive); however, the hemodynamic pattern does not reflect the clinical contribution of the target molecule that might be less or more when in actual clinical use. Septic patients are usually under antibiotic therapy and fluid administration [113], and this might mask the pathogenesis/molecular targets.
- Intervention studies must confirm either mortality rate or CKD development because these are the objectives of the therapy. Attenuating the injury only at one time point does not guarantee that the treatment could prevent patient mortality.
- As far as possible, intervention studies should reflect the clinical setting. Anti-immune therapy might suppress host defenses. Thus, even if it works against sterile inflammation, it might worsen bacteremia and increase mortality.
